# Case Report: A chronic myeloid leukemia patient with e8a2 BCR::ABL1 fusion transcript was successfully treated with Flumatinib

**DOI:** 10.3389/fonc.2025.1670115

**Published:** 2025-09-18

**Authors:** Shanfeng Hao, Yao Zhang, Qing Shao, Lijuan Li

**Affiliations:** Department of Hematology, Tianjin Medical University General Hospital, Tianjin, China

**Keywords:** chronic myeloid leukemia, e8a2 BCR::ABL1 transcript, flumatinib, iron-deficiency anemia, tyrosine kinase inhibitor therapy

## Abstract

The majority of patients with chronic myeloid leukemia (CML) present with BCR::ABL1 transcripts involving the b2a2 (e13a2) and/or b3a2 (e14a2) junctions. However, a small subset of cases exhibit atypical breakpoints. These atypical BCR::ABL1 transcripts are found in approximately 2% of CML patients. It is essential to identify these atypical variants as they respond to tyrosine kinase inhibitor therapy, similar to patients with the more common transcript types. This report described a CML patient with a rare e8a2 BCR::ABL1 transcript variant, who also presented with difficult-to-correct iron-deficiency anemia. The patient was treated with Flumatinib and achieved complete hematologic remission at 1 month, complete cytogenetic remission at 3 months, and major molecular remission at 6 months.

## Introduction

The defining molecular characteristic of chronic myeloid leukemia (CML) is the presence of the BCR::ABL1 fusion gene. This results from forming Philadelphia chromosome t (9; 22) (q34; q11). The BCR::ABL1 transcript is expressed in most patients with CML, with b2a2 (e13a2) and/or b3a2 (e14a2) junctions being the most common. However, there are rare cases in which atypical BCR::ABL1 transcripts are present, accounting for approximately 2% of CML cases (1, 2). The three most frequent uncommon transcript types are e19a2, e13a3/e14a3, and e1a2 (2). The e8a2 BCR::ABL1 transcript is exceedingly uncommon, with only 30 documented cases worldwide to date. In the current study, a 45-year-old female patient with CML in the chronic phase (CP) was identified with a novel e8a2 BCR::ABL1 transcript variant, a rare BCR::ABL1 fusion gene (e8a2), and a combination of difficult-to-correct iron deficiency anemia, in which she responded well to Flumatinib.

## Case presentation

A 45-year-old female patient was admitted to the hospital on January 19, 2024 with a chief complaint of “progressive elevation of white blood cells for 8 months.” Eight months prior to admission, a physical examination revealed elevated leukocyte and platelet (PLT) counts, as well as microcytic hypochromic anemia. The patient’s main concerns included progressively increasing WBC counts, microcytic hypochromic anemia, and thrombocytosis. She also complained of general fatigue, unexplained weight loss, and occasional mild abdominal discomfort. A complete blood count (CBC) revealed the following values: white blood cell (WBC) count of 11.51*109/L, red blood cell (RBC) count of 3.76*1012/L, hemoglobin (HB) level of 76 g/L, and PLT count of 661*109/L. The neutrophil percentage (NEU%) was 63%, mean corpuscular volume (MCV) was 72.1 fL, and mean corpuscular hemoglobin concentration (MCHC) was 280 g/L. Three months before her admission, the patient’s CBC revealed the following data: 4.23*1012/L, WBC count of 24.51*109/L, HB level of 108 g/L, PLT count of 846*109/L, NEU of 73.1%, MCV of 83 fL, and MCHC of 308 g/L. Prior to admission, the patient was treated with an oral polysaccharide-iron complex for iron deficiency anemia, while no specific treatment had been implemented for the underlying hematologic condition. On the day prior to admission, the patient’s leukocyte count increased to 55.92*109/L. Subsequently, the patient sought further consultation at our department. The patient’s medical history was unremarkable with no history of chronic illnesses or prior surgeries. There was no known family history of hematologic malignancies or genetic disorders. The patient did not report any known allergies or prior significant treatments. The patient’s family history was non-contributory with no history of blood disorders or cancers reported in immediate family members. Physical examination revealed a temperature of 36.5°C, a pulse rate of 87 beats per minute, a respiration rate of 16 breaths per minute, and blood pressure of 127/80 mmHg. Abdominal examination noted a mildly enlarged spleen, with its edge palpable 2cm below the rib cage. Upon admission, the patient’s CBC showed a WBC count of 52.35 × 10^9^/L, HB level of 104 g/L, and PLT count of 898 × 10^9^/L. Lactate dehydrogenase (LDH) level was elevated to 680.0 U/L. Tumor marker analysis revealed a glycan antigen level of 199.37 U/mL, indicating an elevated result. Additional laboratory findings included ferritin at 11.89 ng/mL, transferrin at 384.4 mg/dL, and a serum iron concentration of 4.4 µmol/L (decreased). Total iron binding capacity (TIBC) was elevated at 101.5 µmol/L, and unsaturated iron binding capacity (UIBC) was also elevated at 97.1 µmol/L. The erythropoietin level was 29.60 mIU/mL, and the fecal occult blood test (immunoassay) returned a positive result. **Table 1** presents a summary of the CBC values for the patient during different time points. The ‘peak’ notation is used for the values to simplify the presentation and avoid confusion with scientific units. For instance, the WBC count was reported as 11.51 peak/µL initially, 24.51 peak/µL after three months, and 52.35 peak/µL upon admission.

**Table 1:** A summary of the CBC values for the patient during different time points.

Time point	WBC (peak/µL)	RBC (peak/µL)	Hemoglobin (g/L)	Platelets (peak/µL)	NEU% (%)	MCV (fL)	MCHC (g/L)
Initial Admission (01/19/2024)	11.51	3.76	76	661	63	72.1	280
3 months prior (Oct 2023)	24.51	4.23	108	846	73.1	83	308
Day before Admission	55.92	–	–	–	–	–	–
Upon Admission (01/19/2024)	52.35	–	104	898	–	–	–
February 7, 2024 (Post-treatment)	11.16	–	94	825	–	–	–

• “Peak” stands for 10^9/µL for WBC and platelet count, and 10^12/µL for RBC count.

• The table organizes all CBC values in a clear and consistent manner.

Bone marrow aspiration revealed evidence of three-lineage hyperplasia, which may indicate proliferative bone marrow disease. Bone marrow biopsy pathology results showed an increased proliferation of granulocytes and red blood cells, predominantly consisting of mature cells, including megakaryocytes. The cytoplasm of these cells was small, and the nuclei were less folded. There was no significant increase in the number of lymphocytes or plasma cells. Immunohistochemical staining showed occasional positivity for CD117 and CD34, while lysozyme and myeloperoxidase (MPO) were predominantly positive. E-cadherin exhibited minimal positivity, while CD3, CD20, and CD138 displayed slight positivity. Megakaryocytes were positive for CD61.

The leukemia phenotype analysis demonstrated that 6.23% of the abnormal cell population partially expressed CD117, CD33, MPO, HLA-DR, CD13, CD64, CD11b, and CD34, while failing to express cCD3, CD14, CD22, CD79a, CD19, CD10, CD15, or CD7. Histochemical staining revealed that 40% of the cells tested positive for neutrophil alkaline phosphatase (NAP), with a positivity index of 52%. No nucleated erythrocytes were observed, and there was an absence of external iron. Additionally, 28% of the cells tested positive for ferritin, consistent with juvenile erythropoiesis.

Fluorescence *in situ* hybridization (FISH) revealed a 95.5% positive signal for the BCR::ABL1 fusion gene (1R1G1F). Chromosomal karyotype analysis confirmed the presence of the t(9;22)(q34;q11) translocation, resulting in a 46, XX karyotype. Testing for JAK-2, MPL, and CALR mutations was negative. Next-generation sequencing (NGS) revealed secondary mutations, including a c.2851A>G mutation in the SETD2 gene (NM_014159.6), resulting in a p.Asn951Asp substitution. Additionally, tertiary variants were found in the SF1 gene (NM_001178030.1: exon 1: c.182C>T, p.Ala61Val) and the CSF3R gene (NM_156039.3: exon 17: c.2369G>A, p.Gly790Glu). BCR::ABL1 fusion genes P190, P210, and P230 were absent. The rare e8A2 BCR::ABL1 fusion gene was identified, with insertion sequences from an inverted ABL intron 1b (55 bp). There was no evidence of mutations in the ABL kinase region.

The patient was diagnosed with CML with a rare e8A2 BCR::ABL1 fusion gene and PLT count elevation. She was started on targeted therapy with Flumatinib 0.6g daily and hydroxyurea to reduce the tumor load. Over time, the patient’s WBC and PLT counts gradually decreased. By February 7, 2024, routine blood tests showed the following values: WBC of 11.16 × 10^9^/L, HB of 94 g/L, and PLT count of 825 × 10^9^/L. Ferritin was below 30 ng/mL, serum iron was diminished, unsaturated iron-binding capacity was elevated, and blood tests revealed unspecified microcytic hypochromic anemia. Consequently, she was diagnosed with iron-deficiency anemia and continued polysaccharide-iron supplementation. The underlying cause of the iron deficiency, however, remained unclear.

The patient was discharged on February 7, 2024, and continued outpatient treatment with Flumatinib 0.6g once daily. At present, she has been receiving Flumatinib for 10 months with ongoing follow-up. After one month of treatment, routine blood tests indicated normalization of leukocyte and PLT counts, though microcytic hypochromic anemia persisted, which was assessed as complete hematologic response (CHR). Three months into treatment, chromosome analysis revealed no Philadelphia chromosome, while the BCR::ABL1 e8A2 gene remained detectable, confirming a complete cytogenetic remission (CCyR). By six months of treatment, the BCR::ABL1 e8A2 gene became undetectable, marking the achievement of major molecular remission (MMR) (3). **Table 2** presents timeline of key events, laboratory findings, and interventions for the patient with CML (e8a2 BCR::ABL1 fusion).

**Table 2:** Timeline of key events, laboratory findings, and interventions for the patient with CML (e8a2 BCR::ABL1 fusion).

Date/time point	WBC (×10^9^/L)	RBC (×10¹²/L)	HG (g/L)	PLT (×10^9^/L)	NEU%	MCV (fL)	MCHC (g/L)	Key events/interventions
~May 2023 (8 months prior)	↑	↑	↓	↑	–	–	–	Routine physical exam: leukocytosis, thrombocytosis, microcytic hypochromic anemia identified; patient asymptomatic
Oct 2023 (3 months prior)	24.51	4.23	108	846	73.1	83	308	Iron-deficiency anemia treated with oral polysaccharide-iron complex; CBC worsening
Jan 18, 2024 (day before admission)	55.92	–	–	–	–	–	–	Rapid WBC increase, patient seeks further consultation
Jan 19, 2024 (admission)	52.35	–	104	898	–	–	–	Physical exam: mild splenomegaly; labs: elevated LDH (680 U/L), ferritin 11.89 ng/mL, positive fecal occult blood; diagnosis of CML suspected
Jan 20–22, 2024	–	–	–	–	–	–	–	Bone marrow aspiration: three-lineage hyperplasia; Biopsy: increased granulocytes & megakaryocytes; FISH: BCR::ABL1 95.5% positive; Karyotype: t(9;22)(q34;q11)
Jan 23, 2024	–	–	–	–	–	–	–	NGS: SETD2, SF1, CSF3R mutations; BCR::ABL1 e8a2 transcript identified
Jan 24, 2024	–	–	–	–	–	–	–	Treatment initiated: Flumatinib 0.6g daily + hydroxyurea
Feb 7, 2024	11.16	–	94	825	–	–	–	Discharge labs: WBC & PLT improved, microcytic anemia persists; outpatient Flumatinib continued
Mar 2024 (1 month post-treatment)	Normalized	–	–	Normalized	–	–	–	Complete hematologic response (CHR) achieved
May 2024 (3 months post-treatment)	–	–	–	–	–	–	–	Philadelphia chromosome absent; BCR::ABL1 e8a2 still detectable → Complete cytogenetic response (CCyR)
Aug 2024 (6 months post-treatment)	–	–	–	–	–	–	–	BCR::ABL1 e8a2 undetectable → Major molecular remission (MMR)
Present (~Oct 2024)	–	–	–	–	–	–	–	Ongoing Flumatinib therapy; routine CBC & molecular monitoring; iron-deficiency anemia managed with supplementation

## Discussion

The diagnostic work-up for CML in this case included a combination of clinical evaluation, CBC, bone marrow aspiration and biopsy, FISH testing for the BCR::ABL1 fusion gene, and karyotyping. The CBC revealed characteristic findings of leukocytosis, thrombocytosis, and anemia, which prompted further investigation. Bone marrow aspiration showed three-lineage hyperplasia, a hallmark feature of CML, which was further confirmed by the karyotype showing the Philadelphia chromosome t(9;22). The presence of the atypical BCR::ABL1 e8A2 fusion transcript was confirmed using FISH, which is essential for diagnosing atypical CML variants. Genetic analyses, including NGS, were employed to identify secondary and tertiary mutations, such as those in SETD2, SF1, and CSF3R. A notable challenge in the diagnosis was the identification of the rare e8A2 BCR::ABL1 transcript variant. This uncommon transcript, comprising only 0.15% of CML cases, posed difficulties in initial diagnosis due to its rarity and the potential for misidentification as another variant or as a non-CML condition. Additionally, the patient’s concurrent iron-deficiency anemia, which was difficult to correct with oral supplementation, presented a diagnostic challenge in distinguishing between anemia caused by iron deficiency versus anemia secondary to CML-related factors or Flumatinib therapy. The positive fecal occult blood test further complicated the assessment, suggesting possible gastrointestinal blood loss as a contributing factor to the anemia. To clarify the etiology, gastrointestinal endoscopy was considered, although not performed initially. Given the patient’s presentation with leukocytosis, thrombocytosis, and microcytic hypochromic anemia, the differential diagnosis initially included CML, as well as other myeloproliferative disorders like essential thrombocythemia or polycythemia vera, and reactive processes such as inflammation or chronic infections. The detection of the Philadelphia chromosome through FISH testing, along with the BCR::ABL1 fusion gene, confirmed the diagnosis of CML. However, the presence of a rare e8A2 variant necessitated a closer examination of the genetic and clinical implications, as this particular variant might influence the response to treatment. Additionally, the patient’s iron-deficiency anemia was considered in the context of CML-related splenomegaly, bone marrow hyperplasia, and potentially iron malabsorption due to gastrointestinal issues, further complicating the assessment. The patient’s prognosis was initially challenging to assess due to the rare nature of the e8A2 BCR::ABL1 fusion transcript. However, the absence of typical resistance mutations in the ABL kinase region and the initial positive response to Flumatinib, including normalization of blood counts and eventual molecular remission, indicated a favorable prognosis. Follow-up monitoring included monthly CBC to assess hematologic response and BCR::ABL1 gene monitoring via quantitative PCR to track molecular response. At three months, the patient achieved CCyR, and by six months, MMR was achieved, indicating the efficacy of Flumatinib therapy. Continued follow-up is essential to monitor for potential resistance to Flumatinib, particularly with the presence of secondary mutations identified through NGS. The patient’s gastrointestinal health, given the persistent anemia and positive occult blood test, also warrants closer monitoring to determine if an undiagnosed gastrointestinal pathology may be contributing to iron deficiency. Routine follow-up with repeat karyotyping and BCR::ABL1 testing will be necessary to ensure that the patient maintains remission and that no further mutations in the ABL kinase region or BCR::ABL1 gene occur.

The overwhelming majority of patients with CML express the BCR::ABL1 transcript at b2a2 (e13a2) and/or b3a2 (e14a2) junctions. However, some rare cases exhibit atypical breakpoints. A patient with CML was identified with a unique e8a2 BCR::ABL1 transcript variant. Among 4,750 CML patients with detectable BCR::ABL1 gene expression, 4,667 (98.3%) had e13a2/e14a2 (P210) BCR::ABL1 fusion transcripts, while the e8a2 transcript type was observed in 0.15% of cases (2). A review of the literature from PubMed, as of October 2024, revealed 30 reported cases of CML with the e8a2 BCR::ABL1 mutation (2, 4–11). The analysis of these patients included demographic factors, the type of insertion fragment between BCR exon 8 and ABL exon 2, treatment modalities, and therapeutic efficacy. Among these patients, 13 out of 21 were men, 11 out of 18 had elevated PLT counts, and 12 out of 24 had the e8a2 BCR::ABL1 transcript with an insertion of an inverted 55-base pair ABL1 intron 1b sequence. The remaining patients had different insertion fragments. Of the 25 patients who received treatment, 18 were administered tyrosine kinase inhibitors (TKIs), while 7 were treated with hydroxyurea, interferon, or cytarabine. Among the TKI-treated patients, 16 achieved CCyR or MMR, while 2 achieved CHR. One of the two patients who achieved CHR was treated with 400 mg of imatinib daily, achieving sustained CHR after three months (12). However, after 65 months of treatment, the patient developed resistance to imatinib, did not achieve CCyR, and demonstrated no significant reduction in BCR::ABL1 transcript levels. Notably, 18 months after initiating imatinib therapy, an M351T mutation was identified. Moreover, 4 patients who did not receive TKIs treatment still achieved CHR or partial hematologic remission (PHR). Regrettably, one of these patients died 89 months after starting treatment (13). Two other patients who were treated with interferon therapy died (one at 3 years (5) and another at 29 months) (13).

Although the BCR::ABL1 e8a2 mutation is a rare atypical variant, the outcomes and prognosis of patients treated with TKIs do not appear inferior to those with typical mutations. Among the 30 reported cases, only one patient developed resistance to imatinib with the emergence of a T315I mutation; however, CHR was maintained thereafter. This trend was consistent with the present case, in which the patient achieved CHR at 1 month, CCyR at 3 months, and MMR at 6 months under Flumatinib therapy. Notably, numerous CML patients with the e8a2 mutation present with thrombocytosis, as identified in this patient at diagnosis. In addition, she exhibited iron-deficiency anemia, which was managed with iron supplementation. Nevertheless, hemoglobin level was not normalized, and positive fecal occult blood together with a slightly elevated CA199 level was documented during hospitalization. Whether the anemia resulted from chronic occult gastrointestinal bleeding or was associated with Flumatinib therapy remains unclear. To clarify the etiology, further evaluation of iron metabolism parameters and gastrointestinal endoscopy is required. During follow-up, monitoring of BCR::ABL1 e8a2 transcript levels, Philadelphia chromosome status, and ABL kinase region mutations will be continued.

In this study, the e8a2 BCR::ABL1 transcript was identified using NGS analysis, a high-resolution molecular technique that allows for the precise detection of fusion genes and mutations. Additionally, FISH was employed to confirm the presence of the BCR::ABL1 fusion gene, with the typical abnormal signal (+) detected in 95.5% of cells. The reverse transcription polymerase chain reaction (RT-PCR) could also be used to detect and confirm the specific transcript variant in cases of atypical breakpoints such as e8a2, although it was not employed in this study. Moreover, Flumatinib was selected as the treatment option for this patient due to its specific efficacy against CML patients harboring atypical BCR::ABL1 fusion transcripts, such as the rare e8a2 variant. In contrast to traditional first-line TKIs, such as Imatinib, primarily targeting the common P210 BCR::ABL1 fusion gene, Flumatinib has shown to be effective against a broader range of BCR::ABL1 mutations, including those with resistance or atypical fusion points. Additionally, Flumatinib has a favorable safety profile and demonstrated significant therapeutic potential in cases of resistance to other TKIs. Given the patient’s unusual e8a2 BCR::ABL1 fusion, Flumatinib’s broader specificity provided a promising approach, especially since it targets the ABL kinase region more effectively, without the risk of developing resistance as commonly found with other TKIs.

The clinician-assessed outcomes including blood count normalization and molecular response, alongside patient-reported outcomes, help provide a comprehensive understanding of the treatment’s impact. Intervention tolerability was generally good, though the management of iron deficiency anemia remains an area of focus due to its potential impact on patient quality of life and overall treatment adherence. Follow-up diagnostic tests such as serum iron levels, ferritin concentrations, and gastrointestinal investigations will continue to play a key role in guiding treatment decisions and ensuring the long-term success of therapy.

One of the primary strengths of the approach used in this case is the comprehensive diagnostic workup, which incorporated molecular, cytogenetic, and clinical evaluations. This holistic approach allowed for the accurate identification of a rare e8a2 BCR::ABL1 fusion gene in a patient with CML. By combining several diagnostic modalities, we were able to confirm not only the presence of this atypical BCR::ABL1 transcript, but also its specific characteristics, which is essential for personalizing treatment strategies. Another strength lies in the use of targeted therapy with Flumatinib, a second-generation TKI, which has shown effectiveness in managing patients with CML harboring rare BCR::ABL1 mutations like e8a2. The patient’s positive response to Flumatinib, marked by achieving CHR, CCyR, and MMR within a relatively short timeframe, further highlights the importance of utilizing novel TKIs for such rare mutations. This demonstrates that rare BCR::ABL1 mutations should not be regarded as a barrier to effective treatment with TKIs.

However, while the results were promising, limitations also exist in the current approach. Firstly, the short follow-up period restricts our ability to make definitive conclusions about the long-term efficacy and potential resistance to Flumatinib. Although the patient showed early positive responses, the potential for resistance or disease progression after prolonged treatment cannot be ruled out. The rarity of the e8a2 mutation further complicates our ability to predict long-term outcomes, as there is insufficient data to guide expectations for sustained remission in patients with this atypical mutation. Additionally, while the management of iron-deficiency anemia in this patient was initiated with iron supplementation, the underlying cause of the anemia remains unclear. Despite supplementation, the patient’s hemoglobin levels did not normalize, and the positive fecal occult blood test suggested possible gastrointestinal bleeding. This raises questions about whether the anemia was related to prolonged occult blood loss, a side effect of Flumatinib, or another underlying issue. This highlights a significant challenge in managing anemia in patients undergoing TKI therapy, where careful monitoring of iron metabolism and gastrointestinal health is required. Moreover, the scarcity of similar cases in the literature limits our ability to generalize the findings from this case to the broader population of CML patients with rare BCR::ABL1 mutations. The rare nature of the e8a2 fusion gene means that we can only draw from a small body of existing studies, which complicates the development of clear treatment protocols. While our patient responded well to Flumatinib, additional data from other cases are needed to confirm the generalizability of this treatment approach for the e8a2 variant.

## Data Availability

The raw data supporting the conclusions of this article will be made available by the authors, without undue reservation.
